# Artificially Introduced Aneuploid Chromosomes Assume a Conserved Position in Colon Cancer Cells

**DOI:** 10.1371/journal.pone.0000199

**Published:** 2007-02-07

**Authors:** Kundan Sengupta, Madhvi B. Upender, Linda Barenboim-Stapleton, Quang Tri Nguyen, Stephen M. Wincovitch, Susan H. Garfield, Michael J. Difilippantonio, Thomas Ried

**Affiliations:** 1 Genetics Branch, National Cancer Institute, National Institutes of Health, Bethesda, Maryland, United States of America; 2 Laboratory of Experimental Carcinogenesis, National Cancer Institute, National Institutes of Health, Bethesda, Maryland, United States of America; Duke University, United States of America

## Abstract

**Background:**

Chromosomal aneuploidy is a defining feature of carcinomas. For instance, in colon cancer, an additional copy of Chromosome 7 is not only observed in early pre-malignant polyps, but is faithfully maintained throughout progression to metastasis. These copy number changes show a positive correlation with average transcript levels of resident genes. An independent line of research has also established that specific chromosomes occupy a well conserved 3D position within the interphase nucleus.

**Methodology/Principal Findings:**

We investigated whether cancer-specific aneuploid chromosomes assume a 3D-position similar to that of its endogenous homologues, which would suggest a possible correlation with transcriptional activity. Using 3D-FISH and confocal laser scanning microscopy, we show that Chromosomes 7, 18, or 19 introduced via microcell-mediated chromosome transfer into the parental diploid colon cancer cell line DLD-1 maintain their conserved position in the interphase nucleus.

**Conclusions:**

Our data is therefore consistent with the model that each chromosome has an associated zip code (possibly gene density) that determines its nuclear localization. Whether the nuclear localization determines or is determined by the transcriptional activity of resident genes has yet to be ascertained.

## Introduction

Chromosomes assume a non-random and conserved position in the interphase nucleus of higher eukaryotes. It is believed that this localization is correlated with their gene densities. For instance, the gene rich Chromosome 19 is predominantly central, while the gene poor Chromosome 18 is peripherally positioned [1]. Such a pattern is conserved during evolution, is tissue specific [2], [3], and is also maintained when these chromosomes are involved in translocations [1]. Extensive studies in mice also show cell type specific, non-random chromosome arrangements based on both gene density and chromosome size [4]. Together, these data suggest a functional significance of chromosome positioning. However, neither the basis for such an arrangement, nor the nature of its structure/function relationship, has yet been revealed. Thus it remains to be determined how the nuclear distribution of chromosomes correlates with their transcriptional activity.

Non-hereditary forms of colon cancer are defined by a non-random and strictly conserved pattern of chromosomal imbalances. For instance, extra copies of Chromosome 7 can be observed as the sole genomic abnormality in colon polyps [5]. Additional aneuploidies that result in copy number gains of chromosomes and chromosome arms 8q, 13q and 20, and losses of 8p, 17p, and 18q are sequentially acquired at later stages of colon cancer progression, and are faithfully maintained in both metastatic lesions and cell lines derived from the primary tumors [5]–[7]. Through the advent of global gene expression profiling methodologies such as microarrays, it has become possible to identify the consequences of these remarkably conserved chromosomal aneuploidies on the cancer transcriptome. Several recently published studies provide clear evidence that genomic imbalances in tumors directly impact transcript levels [8]–[11].

We have previously described the establishment of a unique model system for systematically studying the consequences of aneuploidy on the cellular transcriptome. This model is based on the introduction of specific chromosomes into karyotypically stable immortalized cells or cancer cells using microcell-mediated chromosome transfer. As in primary tumors, an increase in genomic copy number resulted in increased average transcript levels of genes residing on the aneuploid chromosomes. Additionally, the aneuploidy-induced transcriptional deregulation was found to be neither chromosome nor cell type specific [12]. Thus, aneuploidy does not appear to target only one or a few genes on the affected chromosome, but results in a massive deregulation of a large portion of the transcriptionally active genes.

In the interphase nuclei of normal and tumor cells, the two homologous chromosomes assume a conserved position, largely correlated with their gene densities [1], [13]. Portions of chromosomes involved in translocations were also observed to orient themselves in such a manner as to localize to their inherent positions [1]. We were therefore curious whether an artificially introduced aneuploid chromosome was also capable of finding a position in the nucleus that is similar to its endogeneous homologues. This question, while intriguing of its own accord, was particularly interesting considering the results of our previous study described above [12], which implied that the introduced chromosomes were transcriptionally active. The ability of the introduced chromosome to occupy a specific 3D location would indicate nuclear positioning as a prerequisite for the aneuploidy-induced increase in gene expression. Alternatively, failure to localize to their inherent nuclear space would imply that nuclear positioning of aneuploid chromosomes in cancer cells plays no part in determining their transcriptional activity.

In order to identify the position of aneuploid chromosomes in interphase nuclei, we used 3D-FISH, confocal laser scanning microscopy, and 3D distance measurements on DLD-1 parental and derivative cell lines carrying extra copies of Chromosomes 7, 18 or 19. From a teleological perspective, these experiments further our understanding of the interplay between maintenance of nuclear architecture and genome function. This may impact the way we currently think about treating disease, particularly in aneuploid cancer cells, in which both genomic content and gene expression have been greatly perturbed.

## Results

### Microcell-mediated chromosome transfer of Chromosomes 7, 18 and 19

**Figure 1:**
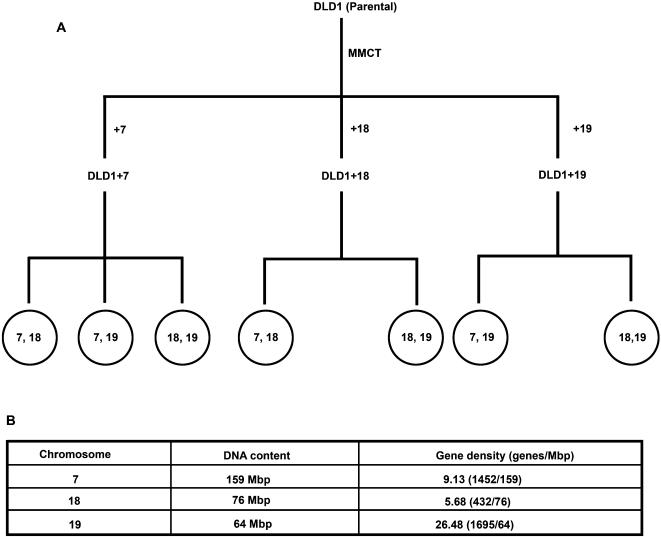
A: Schematic representation of the experimental design. DLD-1 (parental cell line) was subjected to MMCT to generated derivative cell lines DLD-1+7, DLD-1+18 and DLD-1+19. 3D-FISH was performed on each of the derivative cell lines with the probe combinations indicated. B: Table showing comparisons of DNA content and gene density between Chromosome 7, 18 and 19.

As previously reported, a single copy of human Chromosome 7 was successfully introduced into the diploid cell line DLD-1, thereby generating the derivative cell line DLD-1+7 (Figure 1A) [12]. The additional copy of this chromosome directly and significantly increased the average transcript levels of genes residing on Chromosome 7 [12]. This increase was similar upon introduction of Chromosomes 3 and 13 into DLD-1, and was also observed when Chromosome 3 was introduced into normal mammary epithelial cells [12]. The increase in transcript levels is therefore independent of the introduced chromosome and independent of the cell type. For the purpose of this study, we generated two additional cell lines by introducing Chromosomes 18 or 19 into DLD-1 thereby creating the derivative cell lines DLD-1+18 and DLD-1+19, respectively (Figure 1A). We chose these chromosomes because they are of equivalent DNA content (Figure 1B) and because their nuclear positions are distinct and conserved: the gene rich Chromosome 19 is positioned towards the interior of the nuclear space, whereas the gene poor Chromosome 18 is located towards the nuclear periphery [1].

The percentage of cells in a given clone maintaining trisomy for the introduced chromosome, despite continued selection, varied depending on the chromosome transferred and the number of passages. Thus, chromosome positioning measurements were strictly confined to DLD-1 derived cells that were trisomic for the artificially introduced chromosome. While early passage clones of DLD-1+3, DLD-1+7 and DLD-1+13 had a high percentage of trisomic cells and were able to maintain this frequency for up to 12 passages, approximately 20% of the cells in the initial clones of DLD-1+18 and DLD-1+19 were trisomic and this was further reduced at very early passages.

### 3D Distance Measurement of Chromosome Territories

**Figure 2:**
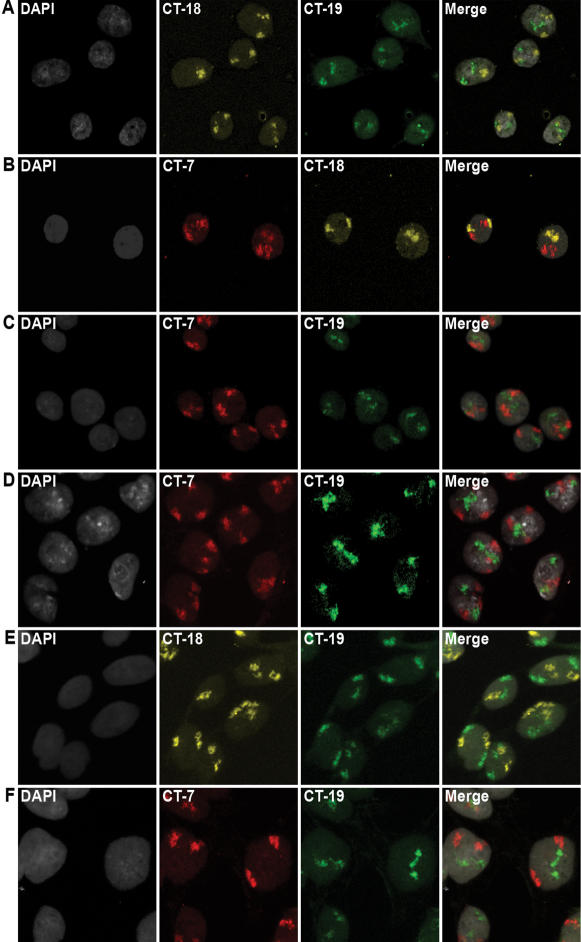
Representative maximum intensity projection of confocal image stacks from DLD-1 parental and derived nuclei. A–C: Parental DLD-1 nuclei. D: DLD-1+7 nuclei. E: DLD-1+18 nuclei. F: DLD-1+19 nuclei. DAPI: DNA counterstain; CT-7: Chromosome 7; CT-18: Chromosome 18; CT-19: Chromosome 19; Merge: merged image of DAPI and chromosome territories.

**Figure 3:**
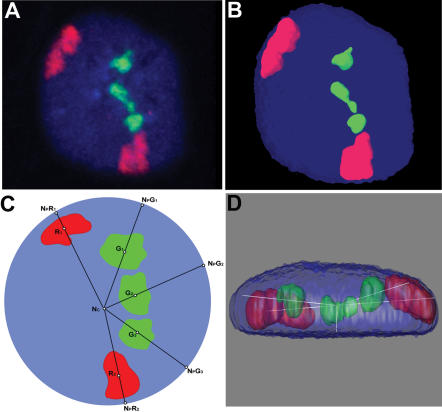
A: Maximum intensity projection of a representative confocal image stack with Chromosome territories 7 (Red, Spectrum orange) and 19 (Green, Rhodamine Green) from DLD-1+19 B: A 3D reconstruction of the nucleus and chromosome territories from the image shown in A (X-Y orientation). C: A scheme adopted for 3D distance measurements of chromosome territories in Red (R_1_ and R_2_) and Green (G_1_, G_2_, and G_3_) from the geometric center of the nucleus (N_c_), to the nuclear periphery (N_P_). Points on the nuclear periphery (eg. N_p_R_1_) are extensions from the nuclear center through the geometric center of the chromosome territory. D: 3D reconstruction in B shown in X-Z orientation.

We first performed dual-color 3D-FISH on morphologically preserved parental DLD-1 nuclei as outlined in Figure 1A. Representative maximum intensity projections of confocal image stacks from each of the three probe combinations (18 & 19, 7 & 18 and 7 & 19) are shown in Figure 2, panels A–C, respectively. In order to objectively evaluate chromosome territory (CT) positioning and to enable a statistical comparison between the parental cell line and its derivatives, 3D image reconstructions were generated using the software Image-Pro Plus (Figure 3). Since we wanted to take into consideration that not all nuclei are completely spherical, we adopted a 3D measurement scheme similar to that of Tanabe et al. ([3] see Methods). The ability to obtain these measurements required the addition of a point on the periphery of the nucleus collinear with the geometric center of the nucleus and the geometric center of the chromosome territory (Figure 3C).

**Figure 4:**
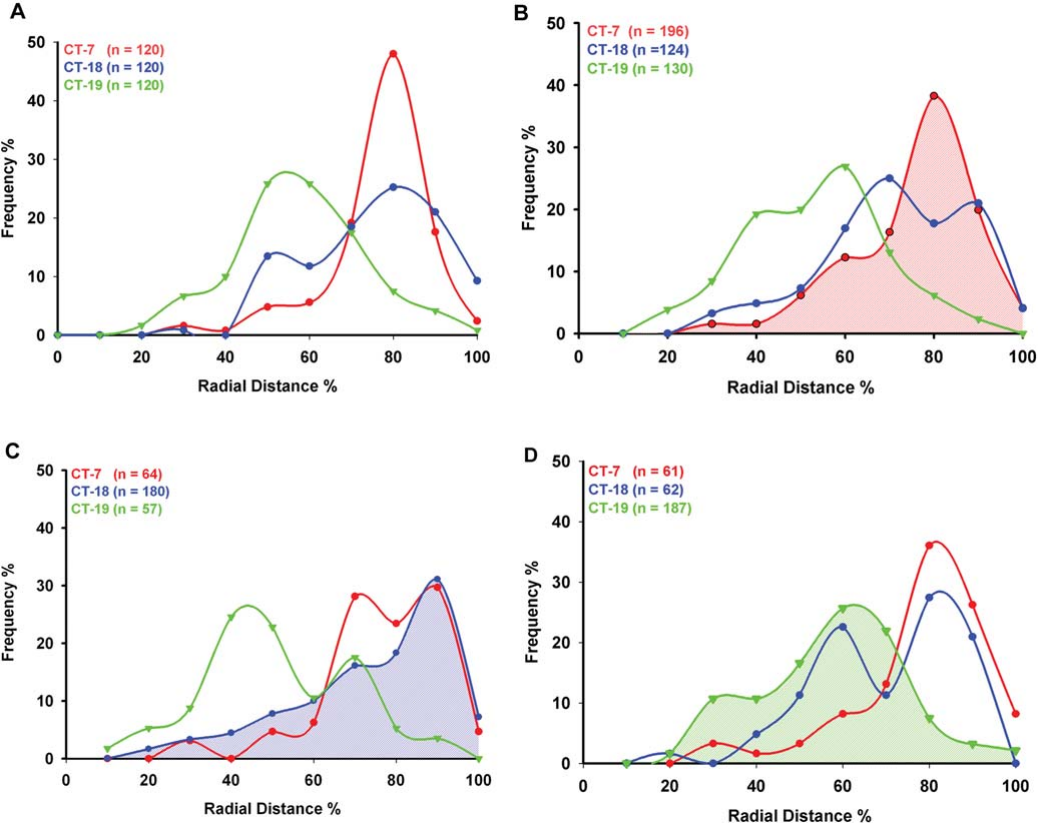
Radial distance measurement profiles of chromosome territories in A: DLD-1 B: DLD-1+7, C: DLD-1+18 and D: DLD-1+19. X-axis: Radial Distance (%); Y-axis: Frequency (%); 0 or origin: center of the nucleus; 100%: nuclear periphery; Red: Chromosome 7; Green: Chromosome 19; Blue: Chromosome 18.

The resulting radial distance measurements were plotted for each chromosome territory. As such, the origin at 0% represents the geometric center of the nucleus, while the nuclear border is considered 100%. Measurements of CT-18 and CT-19 in DLD-1 nuclei show that they are positioned predominantly at a radial distance of 70–80% (peripheral) and 40–50% (central), respectively (Figure 4A). This confirmed previous observations on the positioning of Chromosomes 18 and 19 in DLD-1 [13], and thereby validated our experimental system and analytical procedures. We subsequently performed 3D distance measurements of the intermediately sized, gene poor Chromosome 7 territories. Our results show that CT-7 is radially located in a peripheral position approximately 70–80% from the center of the nucleus (Figure 4A). Similar results were independently obtained using either MIPAV or Imaris software (data not shown).

**Figure 5:**
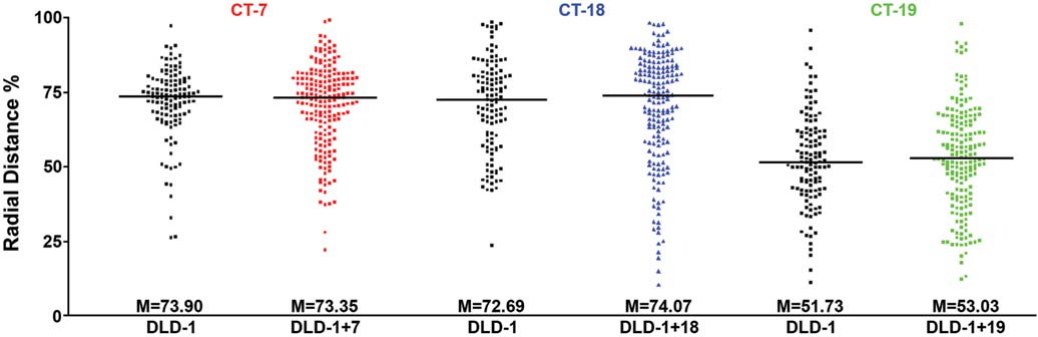
Raw distributions of 3D-distance measurements. CT-7 in DLD-1 & DLD-1+7, CT-18 in DLD-1 & DLD-1+18, CT-19 in DLD-1 & DLD-1+19. X-axis: cell line, Y-axis: Normalized radial distance (%) of chromosome territories from the geometric center of the nucleus.

Having determined the positions of Chromosomes 7, 18 and 19 territories in DLD-1, we analyzed the position of these chromosome territories in nuclei of the three derived cell lines (Figure 2D–F). In all instances dual-color 3D-FISH was performed in various labeling combinations. Our analysis of 3D distance measurements for the three Chromosome 7 territories in DLD-1+7 showed that they assumed a peripheral position in the nucleus at a radial distance of 70–80%, much like in the parental cells (Figure 4B). A comparison of the median values of the radial distance profiles between DLD-1 and DLD-1+7 (73.9 and 73.35, respectively) shows that they are nearly identical with a deviation (Δ_M_ = −0.55) that was not statistically significant as shown by the Mann-Whitney-Wilcoxon test (P = 0.6811) (Table 1; Figure 5).

**Table 1:** Statistical analyses of radial distance measurements of CT-7,18 and 19

	DLD-1 (parental)	DLD-1+7	DLD-1+18	DLD-1+19
CT-7	M = 73.90	M = 73.35	M = 75.97	M = 77.52
	(n = 120)	Δ_M_ = −0.55	Δ_M_ = +2.07	Δ_M_ = +3.62
		P = 0.6811	P = 0.4216	P = 0.0299
		(n = 196)	(n = 64)	(n = 61)
CT-18	M = 72.69	M = 67.10	M = 74.07	M = 66.65
	(n = 120)	Δ_M_ = −5.59	Δ_M_ = +1.38	Δ_M_ = −6.04
		P = 0.0523	P = 0.7820	P = 0.0257
		(n = 124)	(n = 180)	(n = 62)
CT-19	M = 51.73	M = 48.71	M = 43.54	M = 55.02
	(n = 120)	Δ_M_ = −3.02	Δ_M_ = −8.19	Δ_M_ = +3.29
		P = 0.0688	P = 0.0307	P = 0.2677
		(n = 130)	(n = 57)	(n = 187)

Abbreviations:

M: Median

n: number of chromosome territories

Δ_M_: deviation in median values of radial distances from parental cell lines

P:P-value

3D distance measurements were also performed for Chromosome 18 in DLD-1+18, revealing that the three Chromosome 18 territories are positioned at a radial distance of 80–90% (Figures 2E and 4C). The median radial distance values were comparable in the parental and derived nuclei (72.69 and 74.07, respectively; Δ_M_ = +1.38) and were determined not to be significantly different by the Mann-Whitney-Wilcoxon test (P = 0.7820) (Table 1; Figure 5).

Lastly, we determined the nuclear position of Chromosome 19, which was centrally located in the parental DLD-1 cells. 3D reconstructions and distance measurements show that the three Chromosome 19 territories in DLD-1+19 were centrally positioned in the nucleus at a radial distance of 50–60%, equivalent to their position in the parental cell line (51.73 and 55.02, respectively) (Figures 2F and 4D). Again, the difference in median values was not statistically significant (Δ_M_ = +3.29, P = 0.2677) (Table 1; Figure 5). Our studies therefore show that aneuploid chromosomes introduced via microcell-mediated chromosome transfer assume a conserved 3D position in the nucleus indistinguishable from their endogenous homologues.

As mentioned above, we performed dual-color hybridizations with two different chromosome painting probes in the combinations described in Figure 1A. We were therefore not only able to assess the position of the introduced, aneuploid chromosomes, but also to query whether this aneuploidy had any affect on the nuclear position of other chromosome pairs. Upon introduction of an extra copy of Chromosome 7, we observed a tendency for CT-18 and CT-19 to be shifted to a more interior position (Δ_M_ = −5.59 and Δ_M_ = −3.02, respectively). These shifts, however, despite being much greater than those seen in the other derived cells lines, did not reach a statistically significant level (P = 0.0523 and P = 0.0688, respectively) (Table 1). One possible explanation would be that the percent distance measurements for CT-18 and CT-19 in DLD-1+7 had a bimodal distribution and a relaxation of chromosome positioning. The degree of spread as calculated using the weighted-average-Inter Quartile Range (IQR) was 23.84 and 21.08 (for CT-18 and CT-19, respectively) compared to 11.90 for Chromosome 7 (Figure 4B). The introduction of Chromosome 18 had no effect on the position of the two Chromosome 7 territories as they remained at a peripheral position of 70–80% and as such the median radial distance values did not demonstrate a significant shift in position (P = 0.4216). However, the two Chromosome 19 territories were once again shifted more centrally with a radial distance of ∼40% in comparison to ∼55% in the DLD-1 nuclei (Figure 4C). The Mann-Whitney-Wilcoxon test demonstrated that the Δ_M_ = −8.19 was statistically significant (P = 0.0307). The comparison of median radial distance values of CT-7 in DLD-1+19, however, suggested that the position of this chromosome was significantly shifted (P = 0.0299) towards the periphery (73.90 and 77.52; Δ_M_ = +3.62). In addition, Chromosome 18 territories were significantly shifted to a more internal position (72.69 and 66.65; Δ_M_ = −6.04, P = 0.0257).

## Discussion

The systematic exploration of the consequences of chromosomal aneuploidies on gene expression profiles has shown that a direct relationship exists between genomic copy number and transcript levels [8], [10], [14], [15]. In order to generate a model system of chromosomal aneuploidy, we used microcell-mediated chromosome transfer to introduce specific chromosomes into karyotypically stable cells [12]. The results confirmed earlier observations in primary tumors and cancer cell lines, showing a direct impact of chromosomal aneuploidy on resident gene expression levels. This allowed us to study the relationship between aneuploidy and gene expression independent of other cytogenetic abnormalities usually observed in cancer genomes. Having established that the generation of artificial trisomies resulted in a significant increase in average transcript levels of genes on these aneuploid chromosomes, we were now curious as to whether they assume a conserved position in the interphase nucleus. This is an important question because there is firm evidence that native, endogenous mammalian chromosomes occupy specific, conserved 3D positions [3]. For instance, the gene rich Chromosome 19 is localized more centrally, while the gene poor Chromosome 18 territories are positioned more towards the periphery of the nucleus [1]. It is therefore reasonable to surmise a functional relevance of this structural conservation and, as an extension of that, a relationship between 3D architecture and transcriptional activity. With the aim to determine if the increased gene expression correlates with the placement of the introduced chromosome into its conserved nuclear space (e.g., interior for Chromosome 19, and peripheral for Chromosome 18), we performed 3D-FISH on three derived cell lines trisomic for Chromosomes 7, 18 or 19. The DNA mismatch repair deficient colon cancer cell line DLD-1 was used as the recipient cell line. This cell line, as are others with microsatellite instability, is karyotypically stable and diploid. This is advantageous because the position of introduced chromosomes can be assessed without potential confounding effects from other chromosomal aberrations. Here we report that an artificially introduced aneuploid chromosome assumes a non-random and conserved 3D position in the interphase nucleus that is equivalent to the localization of its other two endogenous homologues.

### Positioning of Chromosome 7, 18 and 19 territories

Our analysis of chromosome territories in DLD-1 showed that CT-18 and CT-19 were predominantly peripheral and central, respectively, thus corroborating earlier observations in this cell line [13]. Of note, Cremer et al. reported a smaller difference in the average radial distance between CT-18 and CT-19 in DLD-1 nuclei (∼7.9%) and other tumor nuclei, while our analysis showed ∼18.4% difference between the means of the radial distance measurements of CT-18 and CT-19. However, both studies clearly establish that Chromosome 19 is positioned more towards the interior of the nucleus compared to Chromosome 18. We also show that the gene poor Chromosome 7 is predominantly peripheral in DLD-1, further supporting a gene density based chromosome positioning pattern in both normal and tumor nuclei (Figure 4A) [13], [16].

The primary objective of this study was to assess the relative positioning of the artificially introduced trisomic chromosome compared to its endogenous homologues in all three of the derivative cell lines. We were, however, unable to produce a robust signal with a neomycin FISH probe that would unequivocally denote the introduced chromosome, which is tagged with this selectable marker (data not shown). This was, however, not a major impediment since our statistical analysis did not reveal any significant differences between the localization of any of the three chromosome copies. For instance, all of the Chromosome 7 territories in DLD-1+7 assume a relatively peripheral position in the nucleus (Figure 4B). A similar result was obtained for the 18 (peripheral) and 19 (central) chromosome territories in the nuclei of their respective trisomic cell lines (Figure 4, panels C and D). Thus, there appears to be some mechanism whereby the artificially introduced trisomic chromosomes localize to their innate conserved 3D nuclear position.

A still unexplained finding was the statistically significant, but subtle shift in the median position of Chromosome 19 in the DLD-1+18 cells (Table 1). In addition the mean radial distance between CT-18 and CT-19 increased from 18.4% to 22.69% in these nuclei. One might imagine that extra chromosomes occupying peripheral positions such as Chromosomes 7 or 18 might cause Chromosome 19 to assume an even more central position. Arguing against this reasoning is the fact that the CT-19 shift in DLD-1+7 cells was not significant (Table 1). Additionally, in DLD-1+19, CT-7 is shifted to a more peripheral position while CT-18 is shifted to a significantly more internal position, resulting in a smaller difference in the mean distances between CT-18 and CT-19 (∼11.37%). Thus, while it is relatively easy to understand that the addition of extra chromosome territories into a physically constrained space such as the nucleus would have the potential to induce shifts in the positioning of the other chromosomes, what determines the directionality of that shift is not self-evident. It will be interesting to ascertain how these effects are compounded in aneuploid cancer cells that frequently contain far more than just the one numerical aberration as in our model system. This may possibly be an additional factor in explaining the enormous complexity of gene deregulation in the cancer transcriptome.

We also observed a bimodal distribution of chromosome territories, particularly in the derivative cell lines (Fig. 4A). For example, in a population of DLD-1 nuclei (∼6–8%), CT-18 occupied a more internal position as reflected in a peak at a radial distance of ∼50% from the center of the nucleus. Careful analysis of the raw data did not indicate that this bimodality was a reflection of one chromosome territory in each nucleus behaving differently (e.g., the introduced chromosome), but rather that in some cells all three territories were more central or peripheral relative to the mean. While the relative position of Chromosome 18 and 19 territories is conserved in a wide range of cell types, the degree of this conservation can vary. For instance, some tumor nuclei also showed a decline in the normal radial distribution pattern of CT-18 and CT-19 compared to normal cells. This is particularly apparent for nuclei of the aneuploid colon cancer cell line SW480, where CT-18 and 19 are rather closely positioned [13], suggesting that aneuploidy or additional chromosomal gains could influence the gene density based radial distribution of chromosomes.

### A speculative mechanism of chromosome positioning

It is now clearly established that the positioning of chromosome territories within the 3D space of the interphase nucleus is non-random. This distribution is conserved across different tissues, both normal and malignant, as well as evolutionarily across divergent species [2], [3]. The experiments performed in this study now demonstrate that this non-random and conserved nuclear localization also extends to artificially introduced, aneuploid chromosomes. Thus, such a high degree of conservation lends itself to the idea that there must be some biological implication for the placement of chromosome territories. But how is the functional reorganization of the nucleus established upon reformation of the nucleus after mitosis? Can such a phenomenon be explained mechanistically?

To reiterate the facts: chromosomes with a relatively high gene density occupy a more central position while gene poor chromosomes tend to be localized closer to the nuclear periphery [1]. It is also true that gene rich chromosomes have a higher G-C content. This may partially reflect the presence of CpG islands in gene promoters as well as the preponderance of G-C rich repetitive elements such as Alu sequences that are coincident with coding regions of the genome. In fibroblasts, for instance, an enhanced staining of Alu sequences was found in the nuclear interior [17]. Conversely, the nuclear periphery is enriched for heterochromatin, which has a tendency to be more A-T rich. It is well known that nuclear lamins are critical for the reformation of the nucleus after mitosis. Lamins have also been shown to interact through specific sequences in their tail domain with chromatin and in particular with two of the core histones H2A and H2B [18], [19]. An increased presence of methylated histones, such as tri-H3K27, has been observed near the nuclear periphery [20]. Thus, lamins and variations in nucleosome composition and/or modifications may play a role in the non-random positioning of certain chromosome territories near the nuclear membrane.

What possible factors might be responsible for establishing the above noted features of the nuclear architecture, particularly with respect to the positioning of individual chromosome territories? Perhaps the most intuitive model is one in which each chromosome is identified by a unique “zip-code” that determines where it will reside in the nucleus. One such distinguishing mark could be the unique sequences found in the centromeric or pericentromeric region of each homologue. This hypothesis could be tested experimentally by moving these sequences from one chromosome to another. Fortunately, such events occur naturally via chromosomal translocations. For example, the cancer cell line SW620 contains a der(18)t(17;18) in which material from the gene-rich Chromosome 17 has been translocated to the centromere containing gene-poor Chromosome 18. Despite containing the Chromosome 18 centromere, this derivative chromosome occupies a radial localization similar to the normal Chromosome 17 [13]. This study therefore suggests that chromosome specific centromeres are not the main determinant of chromosome positioning and points more towards the material contained in the chromosome arms.

An alternative to a centromere-specific “zip-code” sequence would be one in which a rather general feature of each chromosome is responsible for placing it in, or excluding it from, certain nuclear regions. In such a model it becomes imperative to explain how features such as gene density, nucleotide composition (G-C versus A-T content), DNA and histone modifications or transcriptional activity are sensed by the re-forming nucleus and are used to establish positioning. Since each of these features is present to a different extent on every chromosome, positioning of territories becomes more probabilistic than definitive. This is consistent with our experimental observations (Figure 4).

As an example, we propose the following scenario as a possible mechanism for establishing the interphase nuclear architecture. Non-transcribed, gene-poor regions of the genome tend to be more heterochromatic, which is predominantly A-T rich. Heterochromatin is established through a combination of DNA and histone modifications that are known to correlate with transcriptional inactivity. Thus, a higher absolute number or concentration of modified nucleosomes, for example tri-H3K27, might make it more likely for a gene-poor chromosome to be snared by lamins attached to the inside of the reforming nuclear membrane. One could then postulate that by default, unsnared G-C rich, gene rich, transcriptionally active chromosomes would have a tendency to be excluded from the nuclear periphery and thus are resolved to occupy a more central nuclear position. In this self-organizing system, the localization of gene-rich sequences in the center of the nucleus is not so much the driving force, but rather the end result of nuclear reformation after mitosis. Others have put forth a self-organizing model wherein the collective transcriptional activity of the genome has been proposed to dictate nuclear architecture based on the physical properties of chromatin and interacting polymerases [21]. Since it is likely that there is very little ongoing transcription in mitotically condensed chromosomes, we would posit that it is not active transcription per se which determines the architecture upon nuclear reformation, but rather the markings of previous transcriptionally active or inactive regions such as DNA and histone modifications.

Gene rich chromosomes are also transcribed more actively. Genome-wide analysis of mRNA expression profiles of the human genome shows that gene dense regions strongly correlate with Regions of Increased Gene Expression (RIDGES) [22], [23]. This would require enrichment or a gradient of increasing concentration of transcription factors or factories towards the nuclear center where there is more transcriptional activity. It would be interesting to determine if such gradients actually exist in the nucleus. If there is a gradient, is it the reason for the non-random distribution of chromosomes or is it established in response to such a nuclear architecture? If a gradient does not exist, is the uniform concentration of transcription factors limiting in gene dense areas of the nucleus with high transcriptional activity? Are the factors in the nuclear interior more transcriptionally engaged than those towards the periphery? Does the higher concentration of heterochromatin in the nuclear periphery restrict not only the accessibility of transcription factors to chromatin, but also impede their ability to traverse the interior of chromosome territories? Answers to these questions will provide significant insight into the role of chromosome positioning in regulating gene expression, a factor which might need to be considered in cells with rearranged genomes.

## Materials and Methods

### Microcell-Mediated Chromosome Transfer (MMCT)

Microcell mediated chromosome transfer (MMCT) was performed as previously described [12]. Briefly, the nuclei of A9 mouse cells maintaining a single human chromosome under G418 selection were fragmented in the presence of cytochalasin-B, sequentially filtered through 8.0, 5.0 and 3.0 µm filters and the purified micronuclei were then fused with the recipient diploid DLD-1 colon cancer cell line using PEG 1500 (Roche, Indianapolis, IN). The derivative cell lines DLD-1+7, DLD-1+18 and DLD-1+19 were generated by isolating single colonies under continuous selection in G418 (200 µg/ml, Geneticin, Invitrogen, Carlsbad, CA). Individual lines were then assessed for the presence of the specific intact transferred human chromosome by 2D FISH with chromosome-specific painting probes on metaphase preparations and the absence of mouse chromosomes as identified morphologically on DAPI stained metaphase preparations.

### Cell Culture

DLD-1 adenocarcinoma cells were grown at 37°C in the presence of 5% CO_2_ in RPMI-1640 media supplemented with 1% L-Glutamine, Penicillin (50 units/ml)/Streptomycin (50 µg/ml) and 10% heat inactivated FBS. The derivative cell lines were grown in the same media supplemented with G418 (200 µg/ml). The cells were plated onto glass chamber slides at an appropriate dilution and allowed to attach overnight at 37°C prior to 3D-FISH analysis.

### Cell fixation and permeabilization

Morphologically preserved nuclei were prepared by a modification of the protocol for 3D-FISH [24]. Cells grown on chamber slides were washed 3 times in 1× PBS for 5 minutes each. The cells were incubated on ice for 5 minutes in CSK buffer (0.1 M NaCl, 0.3 M Sucrose, 3 mM MgCl_2_, 10 mM PIPES adjust pH to 7.4, 0.5% Triton-X-100) and immediately fixed in 4% Paraformaldehyde (PFA) (prepared in 1× PBS (pH = 7.4)) for 5 minutes at RT. The cells were washed in 1.0 M Tris-HCl (pH 7.4) followed by 1× PBS washes 2 times at RT for 5 minutes each. The cells were permeabilized in 0.5% Triton-X-100 (prepared in 1× PBS) for 10 minutes and incubated in 20% glycerol (prepared in 1× PBS) for 60 minutes followed by four freeze-thaw cycles in liquid nitrogen. The cells were washed three times in 1× PBS for 5 minutes each and incubated in 0.1 N HCl for 10 minutes followed by three washes in 1× PBS for 5 minutes each. The cells were stored in 50% formamide (FA)/2× SSC (pH 7.4) overnight at 4°C or until used for hybridization [24].

### 3D FISH

#### Chromosome painting probes

Flow sorted chromosomes 7, 18 and 19 (purchased from M.A Ferguson-Smith and Patricia O'Brien, Univ. of Cambridge, U.K.) were DOP-PCR labeled with either Rhodamine green (Invitrogen) or Spectrum orange (Vysis) as described [25] to generate whole chromosome painting probes. Two differentially labeled chromosome painting probes (1.25 µg) were combined and precipitated with Cot-1 DNA (12.5 µg) in 100% cold ethanol and sodium acetate for 2 hours at −80°C, and centrifuged at 14,000 rpm at 4°C for 30 minutes. The probe was dried under vacuum for 5 minutes and subsequently resuspended in 2.5 µl 100% formamide for 30 minutes shaking in a thermomixer at 37°C and for an additional 30 minutes with 2.5 µl mastermix (50% Dextran sulfate and 2× SSC) at 37°C. The probe was denatured for 5 minutes at 80°C and pre-annealed for 60 minutes at 37°C.

#### Hybridization

120 µl of 70% deionized formamide/2× SSC was applied onto the slide, which was covered with a cover glass and denatured at 78°C for 5 minutes. Immediately following denaturation the excess formamide was shaken off the slide and 5 µl of the probe was spotted to the area of hybridization on which an 18×18 mm^2^ cover glass was placed and sealed with rubber cement. The slides were hybridized for 48 hours in a humid box at 37°C.

#### Detection

The slides were washed in 50% FA/2× SSC, 3 times, shaking for 5 minutes each at 45°C, followed by three washes for 5 minutes, shaking each in 0.1×SSC at 60°C, briefly rinsed in 0.1% Tween20/4× SSC, were counterstained with 4′,6′-diamidino-2-phenylindole (DAPI) for 5 minutes, washed in 2× SSC and mounted in antifade (1,4-phenyline diamine), (prepared in 86% glycerol and pH adjusted to 8.0 with carbonate buffer).

### Confocal Imaging

Confocal images were acquired on a Zeiss LSM 510 NLO confocal system (Carl Zeiss, Thornwood, NJ, USA) with a 100× Plan-Apochromat 1.4 NA oil immersion objective using scan zoom of 2. Z-stacked images were acquired at 512×512 pixels per frame using 8-bit pixel depth for each channel at a voxel size of 0.87 µm×0.87 µm×0.3 µm and line averaging set to 4 collected sequentially in a multi-track, three channel mode.

### 3D reconstructions

Individual nuclei from a merged confocal stack were manually cropped from a given field and 3D reconstructions were performed using Image-Pro Plus (v 5.1) (MediaCybernetics, Silver Spring, MD, USA). Segmentation of the chromosome territories was done by setting a visual threshold of the images in each channel using the original RGB image as a template. In addition, threshold values were independently determined for each channel by using extended depth of field measurements, in which threshold values were typically found to vary between 10–15 units for each channel from the isosurface values. In the 3D-constructor module of Image-Pro Plus, a 3×3×3 lo-pass 3D-filter was applied to each cropped nuclei in all channels to reduce background noise. Surface rendering was performed independently on each channel to obtain the geometric centers of the nuclei (blue channel, DAPI) and the chromosome territories (red channel, Spectrum orange and green channel, Rhodamine Green), respectively (Figure 3).

### 3D-distance measurements

3D distance measurements were performed using Image-Pro Plus (v 5.1). Each nucleus was segmented into 10 equal shells following the model of Tanabe and colleagues [3]. As shown in Figure 3C, the geometric center of the DAPI stained nucleus (N_C_) and the chromosome territories (e.g., Red (Spectrum orange) chromosome territories R_1_, R_2_, etc.) were determined. These geometric centers were connected and extended to a third collinear point on the nuclear periphery (e.g. N_P_R_1_). The relative distance of a chromosome territory from the center of the nucleus was calculated as a percent of the total distance from the center of the nucleus to the nuclear periphery. For example, for the chromosome territory in the Spectrum Orange channel (R_1_):
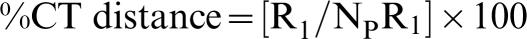



The %CT distance was preferred over the raw distance measurement from the nuclear center in order to scale for variations in nuclear shapes that deviate from a perfect sphere. A merged confocal image stack was subjected to 3D reconstructions (Figure 3A, B) and the distance measurements for each territory were determined. At least 30 nuclei were analyzed for each chromosome combination from DLD-1 and each of the derived cell lines (Figure 1A). Segmentation and 3D-distance measurements were also performed independently using either MIPAV software (CIT, NIH, Bethesda, MD) or Imaris software (Bitplane, Zurich, Switzerland).

### Statistical analyses

The Mann-Whitney-Wilcoxon sum-rank test was used to compare the 3D-distance measurements of chromosome territories between the parental DLD-1 cell line and each of the derivative cell lines. P-values were calculated using Graph pad Prism 3.0 software and were considered statistically significant only when the P-value <0.05 (two sided). Graphical plots of the distance measurements were generated using Sigma Plot 9.0.
